# Case-Based Insights into Enteropathy-Associated T-Cell Lymphoma—Single-Center Experience

**DOI:** 10.3390/hematolrep17050043

**Published:** 2025-08-27

**Authors:** Marija Elez, Lavinika Atanasković, Svetlana Mirosavljević, Mihailo Bezmarević, Dragan Živojinović, Radoslav Romanović, Jelena Djekić, Predrag Krstić

**Affiliations:** 1 Clinic of Hematology, Military Medical Academy, Crnotravska 17, 11000 Belgrade, Serbia; lavinika74@yahoo.com (L.A.); lanacicic@yahoo.com (S.M.); djekic_jelena@yahoo.com (J.D.); predragkrstic@gmail.com (P.K.); 2 Clinic for General Surgery, Military Medical Academy, Crnotravska 17, 11000 Belgrade, Serbia; bezmarevicm@gmail.com; 3 Institute for Pathology, Military Medical Academy, Crnotravska 17, 11000 Belgrade, Serbia; dzivojinovic92@gmail.com; 4 Clinic for Urgent Internal Medicine, Military Medical Academy, Crnotravska 17, 11000 Belgrade, Serbia; rasaromanovic@yahoo.com

**Keywords:** celiac disease, T-cell lymphoma, enteropathy

## Abstract

**Background:** Enteropathy-associated T-cell lymphoma (EATL) is a rare subtype of mature T-cell lymphoma, accounting for fewer than 5% of peripheral T-cell lymphomas, with an aggressive course and poor prognosis. There are two types of this disease based on morphology and immunophenotype: type I, which is often, but not always, associated with celiac disease (classic EATL), and type 2, monomorphic epitheliotropic intestinal T-cell lymphoma (MEITL). Risk factors for classic EATL are poor adherence to a gluten-free diet, advanced age, male sex, and HLA-DQ2 homozygosity. The treatment options include surgery and various chemotherapy regimens with autologous stem cell transplantation, but the outcomes are discouraging, and clinical trials with targeted and biologic therapies are needed. **Case Presentation:** We report three cases of type 1 EATL, all with lethal outcomes, with one patient dying during initial treatment, one dying following several surgical interventions and without waiting to start chemotherapy, and one dying following a good treatment response but with severe infection.

## 1. Introduction

Enteropathy-associated T-cell lymphoma (EATL) is a primary intestinal lymphoma that is often, but not always, associated with celiac disease [1]. This lymphoma type is rare, accounting for less than 5% of peripheral T-cell lymphomas; thus, large clinical studies on this topic are lacking [2]. The term EATL was first used in 1986 by O’Farrelly et al. to describe this type of malignancy [3]. Although the incidence of EATL is low in the general population, it has been shown that it occurs in up to 80% of patients with refractory celiac disease [4]. The incidence of EATL is 0.10 cases per 100,000 inhabitants per year, typically occurring in older age, with a peak incidence in the seventh decade of life. Although uncomplicated celiac disease is twice as frequent in female patients, EATL is more prevalent in males [5]. The 2016 reclassification distinguished EATL from a closely related intestinal lymphoma, monomorphic epitheliotropic intestinal T-cell lymphoma (MEITL). The epidemiological differences between those two subtypes are important as they reflect differences in underlying risk factors, geographic prevalence, and patient populations. Type I (now classified as EATL) is frequently associated with celiac disease and observed in Northern Europe, especially Ireland and Scandinavia, where celiac disease is more prevalent, while Type II, now classified as MEITL, occurs de novo and is predominant in Asia, where celiac disease is rare [6,7]. Both types tend to affect older adults, and MEITL has a higher male/to/female ratio than classical EATL [6,7]. EATL has an extremely dismal prognosis, with a median survival of less than 8 months. This outcome is due to treatment resistance, severe infections, and bowel perforation [8].

## 2. Cases

### 2.1. Case 1

**Figure 1:**
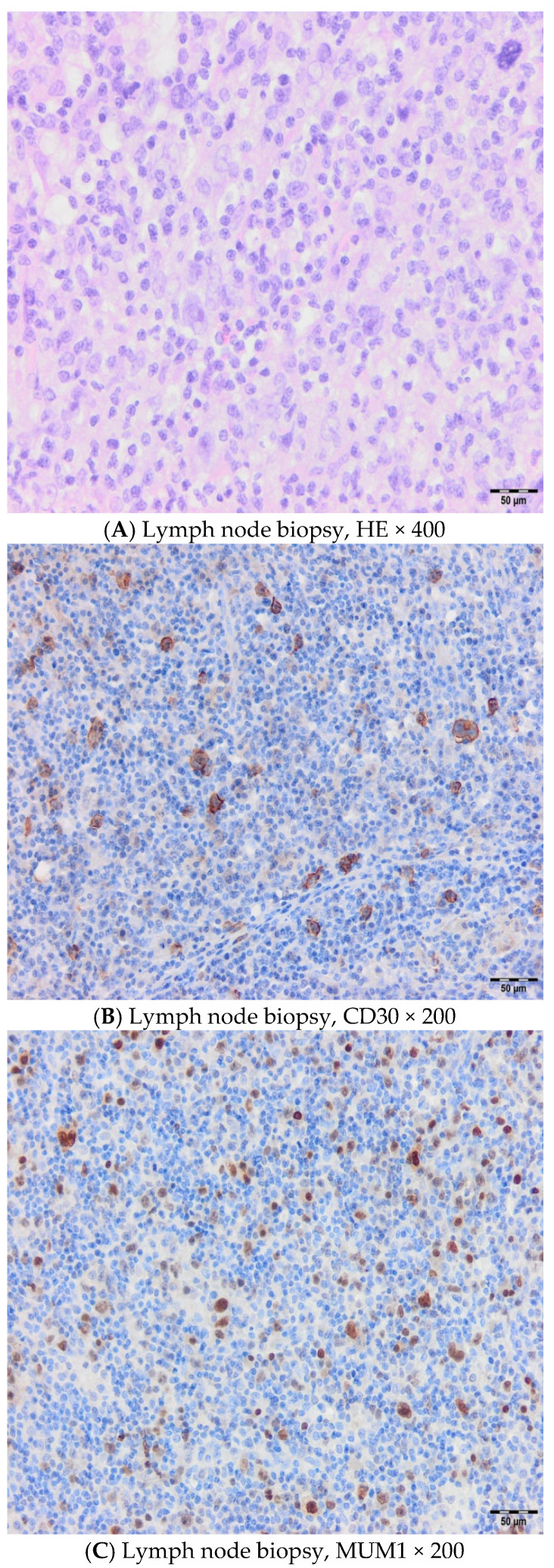
Cervical lymph node biopsy from 2017, when a diagnosis of HL mixed cellularity was established with diffusely altered tissue without lymphoid follicles, mixed cell composition, numerous individual typical and smaller HRS cells without nodular hyalinosis (**A**), CD30 (**B**), and MUM1 (**C**) positive as one of major diagnostic and prognostic marker in B cell lymphomas. Hematoxylin and Eosin (HE); Hodgkin lymphoma (HL); Hodgkin Reed Stemberg (HRS); Multiple myeloma 1 (MUM1).

**Figure 2:**
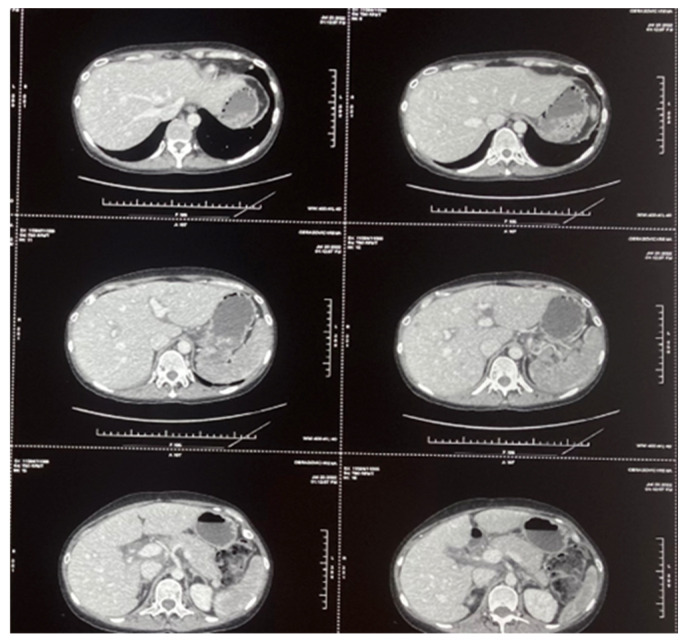
Multi-slice computed tomography (MSCT) revealed liver enlargement and extremely heterogeneous parenchyme with numerous lymphoma infiltrates.

**Figure 3:**
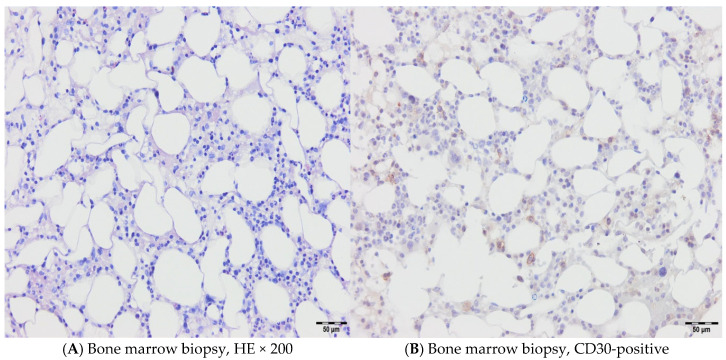
Bone marrow biopsy morphologically showed focal paratrabecular infiltrates of HRS with polymorphic inflammatory background (**A**) and CD30 positivity (**B**) as a key diagnostic marker.

**Figure 4:**
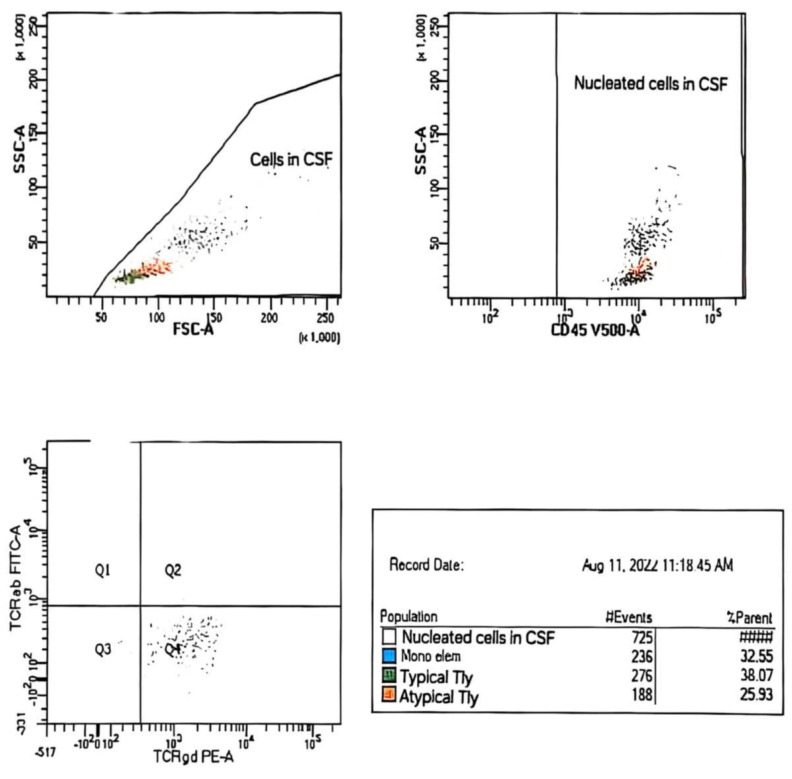
Immunophenotyping of cerebrospinal fluids indicates the presence of 26% atypical T lymphocytes with the characteristics of lymphoma cells. Parallel analysis of peripheral blood showed the presence of relatively small population of lymphoma cells.

A 46-year-old female patient was treated in our hospital for type I EATL. She has previously been treated for Hodgkin’s lymphoma (HL), subtype mixed cellularity (MC) in IV (liver) B-a CS, IPS 2. Her HL was diagnosed in 2017, when she experienced right cervical lymphadenopathy and a suspicious change in the liver. The diagnosis was confirmed via cervical node biopsy, though a liver biopsy was not performed (Figure 1). Complete remission was achieved after six cycles of ABVD regimen (combination of doxorubicin, bleomycin, vinblastine, and dacarbazine). Further follow-up revealed that she was in good health, with complete clinical remission achieved for five years. The patient had a documented COVID-19 infection in January 2022, and, afterwards, the deterioration of her general condition through weakness and malaise was observed. She was occasionally subfebrile and also began to complain of abdominal distension and diarrhea. A complete gastroenterology examination was performed during May 2022 (gastroscopy and colonoscopy), and via gastric biopsy and immunological testing, grade 3 celiac disease was diagnosed. Since the above-mentioned examinations were performed at another institution, we do not have the related images. She was switched to a gluten-free diet, and her stools normalized immediately, but weakness and malaise remained, and liver enzyme levels increased rapidly. A detailed examination was performed (immunological testing, liver magnetic resonance imaging (MRI), liver and marrow biopsy) (Figure 2 and Figure 3), and peripheral T-cell lymphoma (PTCL)—EATL type I—was diagnosed. A Positron Emission Tomography (PET Scan, GE Discovery IQ Gen2 PET/CT, USA) was performed, showing slightly elevated Fluorodeoxyglucose (FDG)uptake in the retroperitoneal lymph nodes and tongue, with a Deauville score of 3. Before the pathological findings arrived, neurological symptomatology in the form of headaches and epileptic seizures developed. The head MRI (MR 1,5 T (2019) and Siemens, Germany) was normal, but a flow cytometry test performed on cerebrospinal fluid indicated the presence of 26% atypical T lymphocytes with the immunophenotypic characteristics of lymphoma cells (Table 1 and Figure 4). Revisions of initial pathology samples (lymph node and bone marrow) taken when she was treated for HL were performed at another institution, and the findings also indicated PTCL, NOS or ALK-negative anaplastic large cell lymphoma (ALCL), but achieving a remission of five years without treatment for such aggressive diseases counters this statement. When a diagnosis of EATL was established with dissemination in the central nervous system, liver and bone marrow, treatment consisted of a combination of immune and chemotherapy, and she has received one cycle of a standard CHOP regimen (cyclophosphamide, doxorubicin, vincristine and prednisone) and two cycles of Brentuximab Vedotin with CHP (cyclophosphamide, doxorubicin and prednisone, vincristine was omitted). Moreover, she received four intrathecal therapies consisting of cytosine arabinoside, methotrexate, and prednisone. A transient improvement in her condition was achieved. Unfortunately, despite complete supportive treatment, symptoms and signs of liver dysfunction were observed, leading to liver encephalopathy. We tried performing plasma exchange to decrease hyperammonemia, but death occurred less than three months after the start of treatment.

### 2.2. Case 2

**Figure 5:**
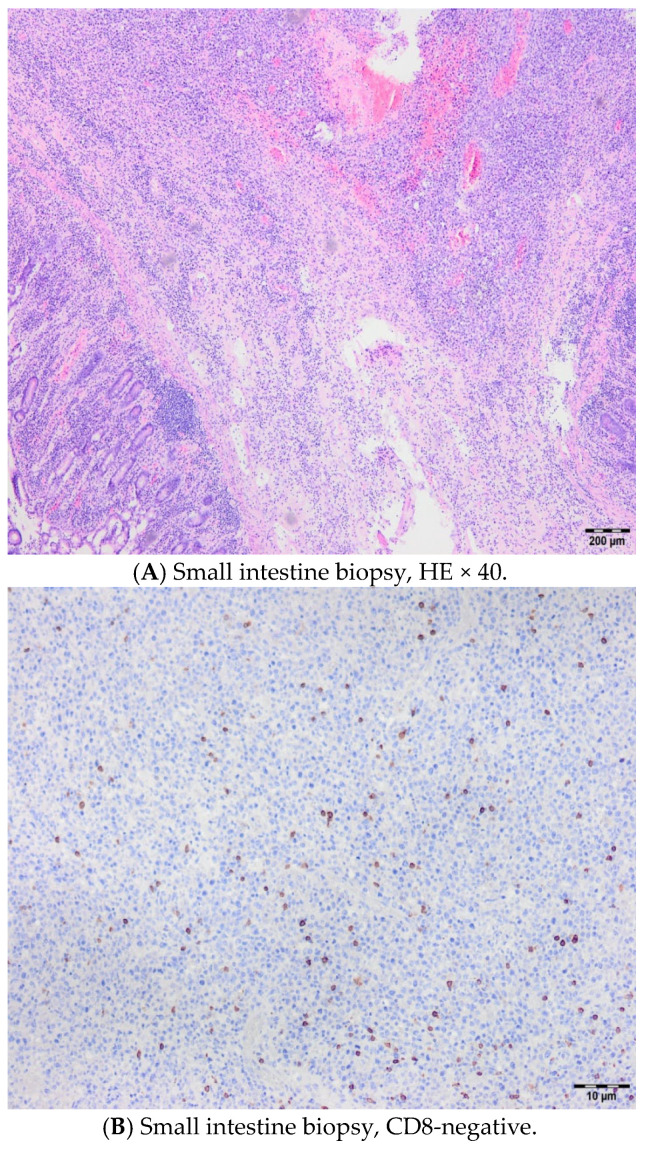

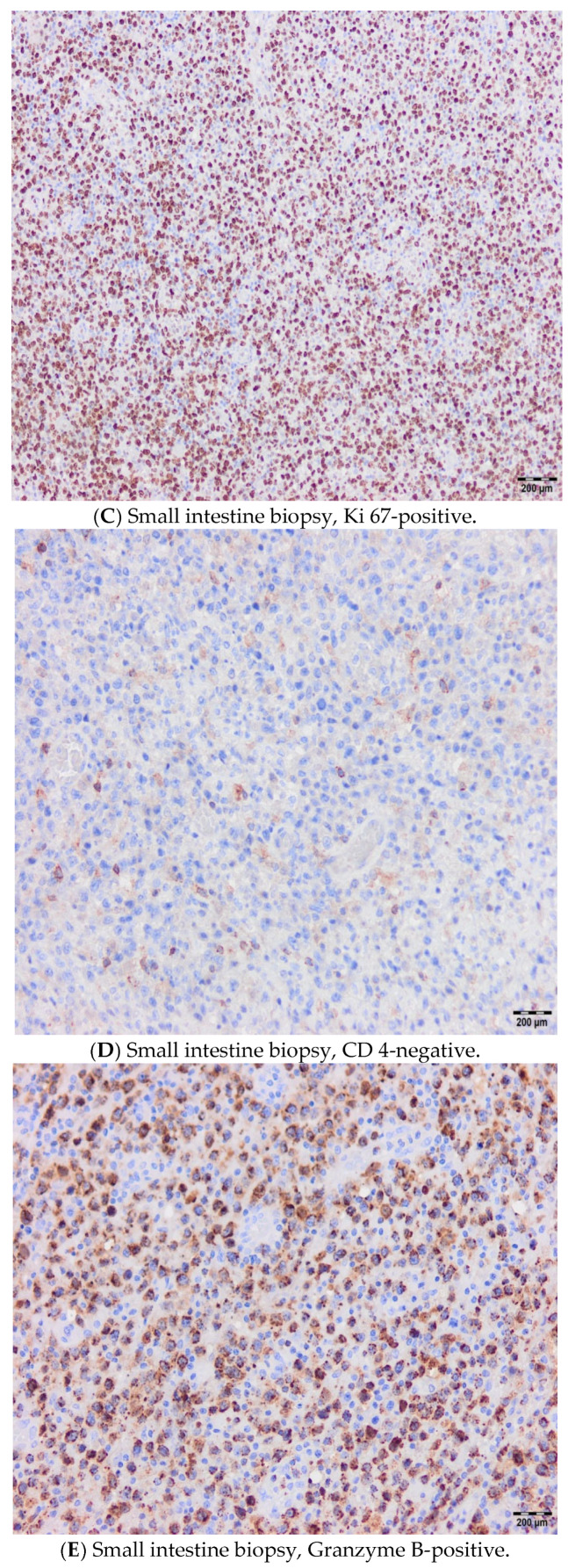
A small intestine biopsy revealing type I EATL; the mucous membrane is atrophic with flattened villi, hyperplastic crypts, and marked intraepithelial T lymphocytosis (**A**), CD8-negative (**B**), Ki67-positive (**C**), CD4-negative (**D**), and Granzyme B-positive (**E**), which corresponds to the severe form of celiac disease Marsh 3c.

A 56-year-old female had gastric pain for three months, and multi-slice computed tomography (MSCT) revealed duodenal infiltration. She was admitted to our institution due to severe abdominal pain, and the clinical findings were consistent with an acute abdominal injury. Immediately after admission to our hospital and adequate preoperative preparation, a surgical intervention was carried out. The intraoperative findings revealed the presence of a large tumor mass of 10 × 10 cm in the region of the left ovary and uterus, infiltrating the lateral abdominal wall. The small intestine was also infiltrated by this mass at the junction of the jejunum and the ileum, causing perforation at that level. Enlarged lymph nodes were seen along the aortocaval vessels all the way to the duodenum, where another perforation was verified. Multiple samples were taken for pathological verification (Figure 5). In the next two months, three more surgeries were performed due to duodeno-cutaneous fistula and duodenal dehiscence. In the meantime, a diagnosis of celiac disease was confirmed, and the patient started on a gluten-free diet. A definite biopsy of the infiltrative mass of the small intestine proved that it was type I EATL, but due to her general condition, no specific therapy was applied, and the patient died from multidrug-resistant Klebsiella pneumoniae sepsis two months after admission to our hospital.

### 2.3. Case 3

A 64-year-old male was referred from another hospital after surgical intervention for a radiologically detected 6 cm mass in the region of the distal jejunum and histologically proven EATL. Afterwards, immunological testing confirmed celiac disease, and the patient started on a gluten-free diet. The clinical manifestations of the disease were fatigue, weight loss, night sweats, pain, and abdominal distension with a palpable mass under the left costal margin. Immediately after admission to our hospital, we started treatment with immunochemotherapy consisting of BV and CHP. He received four cycles of treatment, and complete remission was achieved, according to a PET scan. Unfortunately, the patient had repeated febrile episodes when a fistula was identified between two jejunal whorls, requiring a new surgery. Further treatment was temporarily suspended. The patient died 8 months after diagnosis due to severe infection and sepsis.

## 3. Discussion

Enteropathy-associated T-cell lymphoma (EATL) is a primary intestinal lymphoma usually associated with celiac disease [1]. It is a rare disease, representing fewer than 5% of all peripheral T-cell lymphomas [2]. There are two types of this disease based on morphology and immunophenotype, with both involving the small intestine: type I, which is often related to celiac disease (classic EATL), comprises 80 to 90% of all EATL cases, histologically presents with pleomorphic large or medium-sized cells with prominent inflammatory niches, and is most often CD4- and CD8-negative, and type 2, which is a monomorphic epitheliotropic variant, known as MEITL due to a revised 2016 classification, and most often CD 8-positive [6,7,9].

Common clinical manifestations of classic EATL are abdominal pain, weight loss, diarrhea, bowel obstruction, and perforation of the small intestine. Lymphoid infiltrates are usually multifocal, causing plaques, ulcerations, strictures with enlarged mesenteric lymph nodes, and large tumor masses. Dissemination in the liver, spleen, lungs, and skin is also common, while bone marrow involvement is unusual [10]. EATL has a much worse prognosis when compared with other T-NHL variants, such as T-cell lymphoblastic lymphoma, anaplastic large cell lymphoma, adult T-cell lymphoma/leukemia, angioimmunoblastic T-cell lymphoma, extranodal NK/T-cell lymphoma, and peripheral T-cell lymphoma (not otherwise specified) [11]. 

Various treatment regimens have been applied for treating EATL during the past fifteen years, including either chemotherapy alone, mainly through a CHOP regimen, or in combination with surgical resections and, later, autologous stem cell transplantation, though outcomes are poor. EATL has an extremely poor prognosis, and according to one multicenter analysis, 82% of patients have typically died after a median of 7.4 months [12]. Such an outcome is explained by treatment resistance, severe infections, and bowel perforation [8]. Risk factors for classic EATL are poor adherence to a gluten-free diet, advanced age, male sex, and HLA-DQ2 homozygosity [13]. The most favorable outcomes are seen in patients who undergo autologous stem cell transplantation, with 5-year survival reaching up to 60% [14,15]. The Scotland and Newcastle Lymphoma Group (SNLG) prospectively collected data on all patients with a new diagnosis of EATL made between 1994 and 1998, treated with ifosfamide, vincristine, etoposide/methotrexate following autologous stem cell transplantation (IVE/MTX-ASCT regimen), aged 18 years or older, and ability to tolerate high-dose treatment. Treatment results of IVE/MTX-ASCT were compared with the results of the historical control group treated with conventional anthracycline-based chemotherapy, showing significantly improved remission rate, five-year progression-free survival (PFS), and overall survival (OS), and lower death rates in the IVE/MTX-ASCT group [16]. While EATL was not a primary focus of ECHELON-2, global, randomized, double blind, phase III registrational trial designed to evaluate the efficacy and safety of BV + CHP in patients with CD30-positive PTCLs, the rationale for extrapolating BV + CHP to EATL includes proven better overall response rate (ORR), PFS and OS in all CD30 + PTCLs and based on this trial, NCCN guidelines now include BV + CHP for CD30 + PTCLs, which can encompass CD30 + EATL [17]. There are some data about transient favorable responses to therapy based on a monoclonal antibody that targets CD52 (almetuzumab) [18,19]. Moreover, some clinical activity has been achieved with cladribine, a synthetic purine nucleoside with cytotoxic effects, and romidepsin, a histone deacetylase inhibitor [20,21] (Table 2).

Our experience correlates with the literature with respect to the clinical course and poor outcomes. Two out of three of our patients were females, although according to the literature, the disease occurs more often in males. Also, more than 95% of celiac disease patients carry HLA-DQ2, while the percentage of the general population with HLA-DQ2 is much lower, but none of our patients underwent molecular testing, and we proved EATL diagnosis with biopsies. Immunohistochemistry (IHC) is essential for EATL identification and is performed in all our cases, which include CD3, CD7, CD8/CD4, CD30, CD103, TIA-1, Granzyme-B, Perforin, and Ki-67. The imaging implied MSCT (>16-slice), MRI (T1, T2, STR, DWI sequences), ultrasound, and PET scan (uses 18F-FDG). According to our knowledge, there are no data on the development of EATL after previous treatment for Hodgkin’s lymphoma, as noted in our first case. Despite the revision of the initial pathology samples (lymph node and bone marrow) taken at another institution indicating that patient initially also had PTCL-, NOS- or ALK-negative ALCL, achieving a remission of five years without any treatment for such an aggressive disease undermines this claim and we can conclude that it is only hypothesis since both Figure 1 and Figure 3 histologically support the diagnosis of HL. Up to 50% of patients die before starting active treatment, which was also the case for our second patient, who underwent four surgical interventions and died from sepsis. Using a novel treatment approach with a combination of BV and CHP for our third case led to complete remission, but due to a severe infection caused by a friable intestine and fistula, further treatment had to be delayed, and the patient later died from sepsis. Unfortunately, our two patients did not wait for the autologous stem cell transplantation, which might improve the disease course and outcome, due to a lack of chemosensitivity in the first, i.e., poor general condition in the second case.

To improve the outcomes of patients with EATL, clinical trials with targeted and biologic therapies are necessary. To better understand mutational landscapes and gene polymorphisms, Manso et al. analyzed a series of EATL and MEITL cases and identified possible therapeutic targets. The majority of EATL patients exhibited mutations in DNA repair genes (TP53), followed by NOTCH, VEGF, and PI3K/AKT signaling pathways [22]. Moreover, the therapeutic efficacy levels of drugs targeting JAK2-STAT3 gain-of-function mutations, such as ruxolitinib, which inhibits JAK1 and JAK2, and abrocitinib, which specifically inhibits JAK1, were preclinically tested [23]. The effects of these two drugs were compared with the non-resorptive corticosteroid budesonide and the proteasome inhibitor bortezomib, which can interfere with the STAT3 signaling pathway [23]. Ruxolitinib and abrocitinib were shown to trigger apoptosis, reduce proliferation, and simultaneously block STAT3 phosphorylation. Hence, inhibition of the JAK1-STAT pathway holds promise as a therapeutic approach to prevent neoplastic progression to EATL and improve the prognosis of the disease [24]. Celiac disease is associated with chronic intestinal inflammation, leading to the overexpression of IL-15 and proliferation of intraepithelial lymphocytes. Blocking IL-15 with Janus kinase inhibitors has shown promising results in animal models and might also be a future treatment approach in humans [25]. EATL is an exceptionally rare subtype of PTCL, making large-scale studies and clinical trials difficult. Most data come from retrospective case series with small sample sizes, limiting the strength of evidence and generalizability. Also, comprehensive genomic profiling is lacking, hindering the identification of biomarkers and targeted therapies. International cooperation is essential to pool cases, perform prospective trials, and validate treatment protocols.

## 4. Conclusions

It seems that adequately monitoring patients with diagnosed celiac disease and the early detection of pre-lymphoma lesions might be crucial [26]. Twenty years ago, it was proven that strict adherence to a gluten-free diet is the most important factor in reducing the risk of developing celiac disease complications [27]. It is obvious that, as in other hematological malignancies, a better understanding of the molecular features in EATL would be beneficial for further optimizing diagnostic and therapeutic options [28]. Whilst tissue biopsy remains the diagnostic gold standard, but often difficult to perform, molecular heterogeneity might be analyzed from the liquid biopsy through the analysis of circulating tumor DNA (ctDNA), which is a non-invasive tool offering genetic profiling and thereby a future perspective for various lymphoma types. Liquid biopsy could potentially transform both diagnostic and monitoring strategies in EATL. After diagnosis of celiac disease, this could help detect clonal T-cell populations or early genetic aberrations before overt lymphoma develops. Using ctDNA allows detection of minimal residual disease, treatment response, and reveals key mutations (e.g., JAK1, STAT3, or SETD2) even when tissue samples are unavailable or insufficient. Possible biomarkers for EATL via liquid biopsy might be JAK1/STAT3 mutations, SETD2 mutations, and T-cell receptor (TCR) clonality, but their clinical utility in EATL remains largely exploratory [29].

## Figures and Tables

**Table 1:** A flow cytometry test with the immunophenotypic characteristics of lymphoma cells.

**Sample/Method**	CSF,~2 mL/WLLyW/ Multparameter/ flow cytometry/ BD FACSCanto II (4-2-2)		
**Description of CSF**	Clear; no admixture of red blood cells in sediment		
**Analysed combination of Ag**	1. mCD3/CD16/CD56/CD4/CD19/CD8/CD45 2. TCRαβ/TCRγδ/CD4/CD7/CD8/CD14/mCD3/CD45 3. CD2/CD5/CD4/CD7/CD8/mCD3/CD45 4. CD57/CD30/CD4/CD7/CD8/mCD3/CD45		
**No. of analyzed cells ep**	725 nucleated cell in CSF		
**Population of cells in CSF**	Gating strategies	% SC/number of cells	
**Nucleated cells**	CD45^+^/SSC	100% (725 c)	
**Mono elements**	CD14^+high^ CD45^+high^/SSC^medium^	33% (236/725 c)	
**Typical T-Ly**	mCD^+med^ CD4^+^ CD8^+^ CD7^+het^ TCRαβ^+^ TCRγδ CD45^+high^/ SSC ^low^	38% (276/725 c)	
**Atypical T-Ly**	mCD3^+low^ CD2^+high^ CD7^+bright^ TCRγδ^+low^ CD30^+low^ CD4^+^ CD8^+^ TCRαβ^+^ CD57^+^ CD45^+high^/SSC^low^	26% (188/725 c)	
**Population of Atypical T-ly (188c–725c)**			
**Antigen**	**% positive cells**	***Antigen***	**% positive cells**
CD45^+high^	100%	CD4	0%
mCD3^+Low^	100%	CD8	0%
CD7 ^+bright^	100%	CD5	0%
CD2 ^+high^	100%	CD16	0%
CD30 ^+low^	64%	CD56	0%
TCRγδ^+low^	91%	CD57	0%
TCRαβ	0%	CD19	0%
Ag^+^ = partial exp. – exp. on subpop. cells			
**Conclusion:** Analysis of CSF sediment revealed the presence of an atypical population of T-Ly with immunophenotypic characteristics corresponding to lymphoma cells (~26% NC → 188c/725c). Parallel analysis of peripheral blood cells revealed the presence of a relatively small population of lymphoma cells (mCD3^+low^ CD2^+high^ CD7^+bright^ TCRγδ^+high^ CD30^+het^ CD4^+^ CD8^+^ TCRαβ^+^ CD45^+high^ / SSC^low^, ~0.9% Le).			

Cerebrospinal fluid (CSF); Antigens (Ag); nucleted cells NC, instead of SC (% SC); lymphocyte (T-Ly); nucleated cells (NC).

**Table 2:** Major completed and ongoing clinical trials for EATL.

Trial Name/ID	Intervention(s)	Status	Design/Notes	Key Findings
NCT03217643 (EATL-001)	BV + CHP following HDT + ASCT	Phase 2/completed	14 newly diagnosed CD30 + EATL type I	ORR 79%, CR 64%, 2y PFS 63%, OS 68%
SNLG	IVE/MTX + ASCT	Retrospective/completed	26 transplant eligible EATL	5y PFS~52%, OS~60%, versus anthracycline- only 7 mo OS
NCT00697346	Alisertib (MLN8237)	Phase 1/completed	Advanced hematologic malignancies, incl. EATL	EATL specific results not detailed
NCT00901147	Bortezomib + panabinostat	Phase 2/completed	R/R PTCL incl. EATL	No separate EATL data reported
NIH CD30 CAR-T trial	CD30-CART cells post- fludarabine+ cyclophosphamide	Phase 1/recruiting	CD30+ lymphomas incl. EATL	Ongoing
NIH/Mayo Clinic Nivolumab	Nivolumab (anti-PD-1)	Phase 1/recruiting	PTCL incl. EATL	Ongoing
AMG714 for RCDII	Anti IL-15 monoclonal antibody	Phase 2a/completed	Type II refractory celiac disease (pre-EATL)	Halted malignant IEL progression, relevant as EATL prevention
NCT02588651	BV monotherapy	Phase 2/recruiting	CD30-low mature T-cell lymphomas, possibly EATL	Ongoing, may include EATL subgroups
ASTX660	ASTX660, Duvelisib combos	Phase I/II/active/terminated	R/R PTCL, some EATL	EATL data pending

Brentuximab + Vedotin (BV); Cyclophosphamide + doxorubicin and prednisone (CHP); ifosfamide, vincristine, etoposide/methotrexate following autologous stem cell transplantation (IVE/MTX + ASCT); Overall Response Rate (ORR); Complete Remission (CR); Progression Free Survival (PFS); mo (month); overall survival (OS); Peripheral T-cell lymphoma (PTCL); intraepithelial lymphocytes (IEL).

## Data Availability

No new data were created or analyzed in this study. Data sharing is not applicable to this article.
